# Mayer-Rokitansky-Küster-Hauser syndrome complicated by hematometra and hematosalpinx treated with laparoscopic uterine drain placement: a case report

**DOI:** 10.1093/jscr/rjaf797

**Published:** 2025-10-07

**Authors:** Uma V Mahajan, Marla Sacks, Carlos Reck, Varag Abed, Harry Zinn, Francisca T Velcek

**Affiliations:** Department of General Surgery, Division of Pediatric Surgery, State University of New York, 445 Lenox Road, Brooklyn, NY 11226, United States; Department of General Surgery, Division of Pediatric Surgery, State University of New York, 445 Lenox Road, Brooklyn, NY 11226, United States; Department of General Surgery, Division of Pediatric Surgery, State University of New York, 445 Lenox Road, Brooklyn, NY 11226, United States; Department of General Surgery, Division of Pediatric Surgery, State University of New York, 445 Lenox Road, Brooklyn, NY 11226, United States; Department of Radiology, State University of New York, 445 Lenox Road, Brooklyn, NY 11226, United States; Department of General Surgery, Division of Pediatric Surgery, State University of New York, 445 Lenox Road, Brooklyn, NY 11226, United States

**Keywords:** Mayer-Rokitansky-Küster-Hauser syndrome, Mullerian duct anomaly, uterovaginal malformation, hematometra, cyclic abdominal pain, case report

## Abstract

Mayer-Rokitansky-Küster-Hauser (MRKH) syndrome is a reproductive tract malformation occurring in ~1 in 5000 female births. It is characterized by congenital agenesis or marked hypoplasia of the Mullerian ducts derivative structures. About 3% of MRKH patients have a functioning uterus. A 12-year-old otherwise healthy female presented with persistent cyclic severe right lower quadrant pain. She was found to have MRKH syndrome with a right-sided functioning uterus and hematometra and ipsilateral hematosalpinx. She subsequently underwent diagnostic cystoscopy, vaginoscopy, and pelvic laparoscopy. Laparoscopic drainage of the hematometra and hematosalpinx, and hysterostomy catheter placement and uterine fixation to anterior abdominal wall were performed. Symptoms fully resolved post-operatively. A computed tomography scan 2 weeks later demonstrated resolution of the hematometra and significant improvement in the hematosalpinx. MRKH syndrome with a functioning uterus may present as cyclic abdominal pain in adolescent female patients. Laparoscopic uterine drain placement is a management option.

## Introduction

Mayer-Rokitansky-Küster-Hauser (MRKH) syndrome is a reproductive tract malformation in females characterized by congenital aplasia or severe hypoplasia of the Mullerian duct derivative structures, including the proximal third of the vagina, cervix, uterus, and fallopian tubes. It can be associated with kidney and skeletal malformations [1]. MRKH syndrome affects ~1 in 5000 live female births [2]. The majority (95%) of patients have bilateral rudimentary uterine structures joined with a fibrous band, while a minority show no (3.5%) or unilateral (1.5%) rudimentary uterine structures [3]. Of patients with uterine structures, only 2.6% have a three-layer uterine differentiation with hematometra and/or ipsilateral hematosalpinx [3]. We present a rare case of a patient who was found to have MRKH syndrome with a unilateral uterus with hematometra and ipsilateral hematosalpinx.

## Case presentation

A 12-year-old female with no significant past medical history, born full term with no gross physical abnormalities, presented to the emergency department with one week duration of severe right lower quadrant pain. This episode was preceded by similar cyclic right lower quadrant abdominal pain for several months. She had never had menstrual vaginal discharge. On examination, her vital signs were within normal limits, and she was of age-appropriate height and weight. Tanner stage 4 breast development and pubic hair were present.

**Figure 1:**
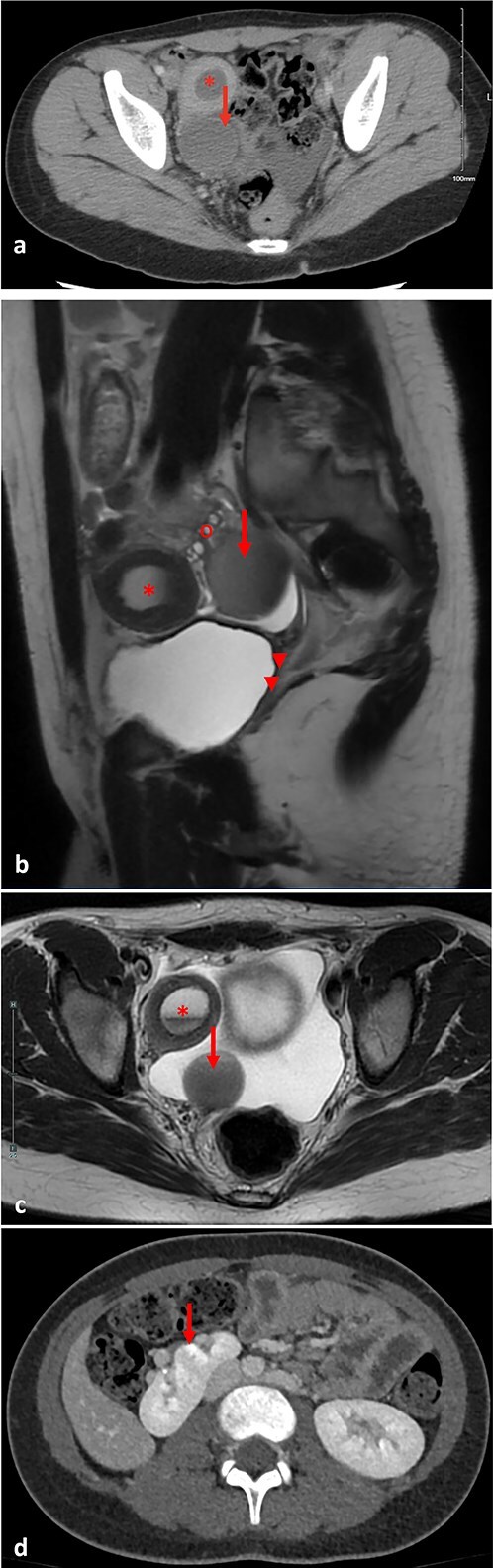
(a) Pre-operative axial computed tomography imaging, demonstrating a right uterine horn with hematometra (^*^) and a right adnexal cystic structure representing hematosalpinx (arrow). (b) Pre-operative pelvic parasagittal T2 weighted MRI, demonstrating a right uterine horn with hematometra (^*^), a right adnexal cystic structure representing hematosalpinx (arrow), normal right ovary (o), cervical aplasia, and vaginal atresia (arrowheads). (c) Pre-operative pelvic axial T2 weighted MRI, demonstrating a right uterine horn with hematometra (^*^), and a right adnexal cystic structure representing hematosalpinx (arrow). (d) Pre-operative abdominal axial T2 weighted MRI, demonstrating a right supernumerary kidney (arrow).

An abdominal ultrasound was done to rule out appendicitis and showed free fluid in the right lower quadrant. A follow-up pelvic ultrasound showed a complex right adnexal cystic structure. A computed tomography abdomen/pelvis (CT AP) was performed and showed an abnormally positioned uterus in the right hemipelvis with hematometra, no identifiable cervix, a right adnexal cystic structure measuring 3.7 cm, free fluid in the pelvis, and a supernumerary right kidney fused to the native right kidney. This constellation was suggestive of a Mullerian duct anomaly. Pelvic magnetic resonance imaging (MRI) demonstrated a right unicornuate uterus with hematometra with an absent left horn, a right dilated hematosalpinx measuring 3.8 cm, a left ovary displaced to the anterior left lower quadrant, cervical aplasia, and vaginal atresia consistent with a Mullerian duct anomaly (U5a C4 V4) (Fig. 1).

**Figure 2:**
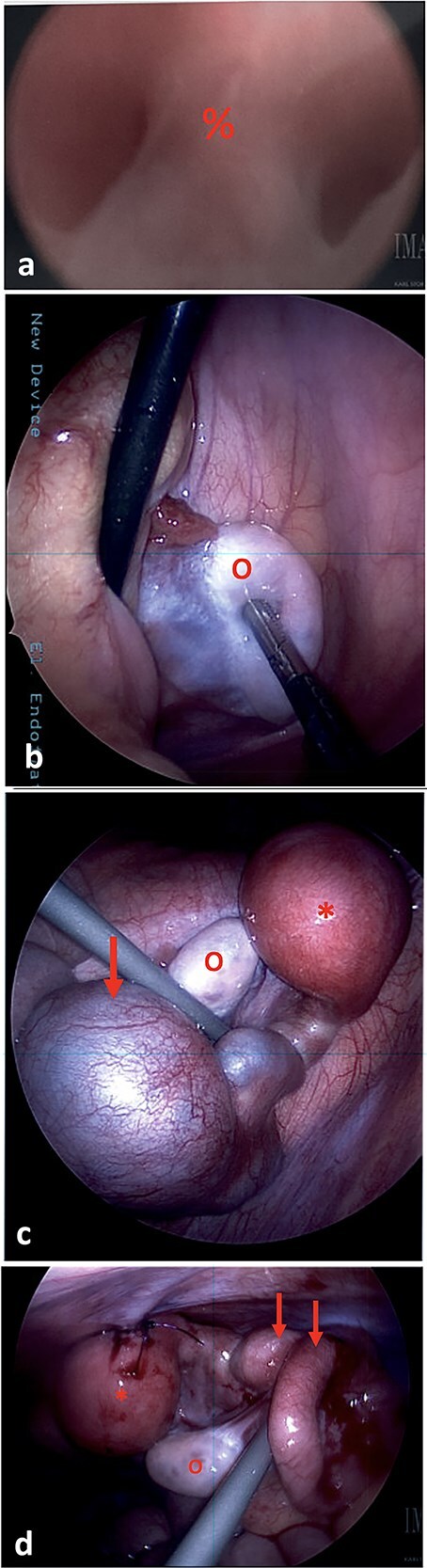
(a) On vaginoscopy, a central septum (%) was noted, suggestive of a longitudinal vaginal septum or hemi-vaginas. (b) Laparoscopic photograph showing the left ovary (o) located in the left lower pelvis, not attached to any tubal structure. (c) Laparoscopic photograph demonstrate a right-sided hypertrophic uterus (^*^), significantly dilated and firm due to retained blood, and a markedly dilated and tortuous right fallopian tube (arrow). The right ovary appeared normal (o). (d) The uterine dome (^*^) was sutured to the abdominal wall. The right fallopian tube was decompressed after aspiration (arrows).

The patient was admitted for a diagnostic cystoscopy, vaginoscopy, and pelvic laparoscopy. An examination under anesthesia demonstrated normal external genitalia, with normal vulvar, urethral, and anal anatomy. A small vaginal dimple was present in the introitus, consistent with vaginal atresia. Vaginoscopy was attempted, but could only be advanced 0.3 cm. A central septum was noted (Fig. 2a). Cystoscopy was unremarkable.

During diagnostic laparoscopy, 50 ml of clear serous fluid was aspirated from the pelvic cul-de-sac. A left ovary was present in the left lower quadrant, but no left sided uterine or tubal structures were identified (Fig. 2b). On the right side, a hypertrophic hemiuterus was visualized, significantly dilated and firm due to retained blood (hematometra) (Fig. 2c). The right fallopian tube was markedly dilated and tortuous with a cystic dilation at the mid portion consistent with hematosalpinx. The right ovary appeared normal. No intrabdominal vaginal structures were noted.

The cystic segment of the fallopian tube was laparoscopically punctured and 60 ml of dark blood was aspirated. The uterine fundus was separately punctured and 40 ml of dark blood was aspirated, consistent with hematometra. A 10Fr silicone Foley catheter was inserted into the uterine dome using a hook cautery device. The balloon was inflated, and the uterus was affixed to the abdominal wall with 2-0 PDS sutures (Fig. 2d).

**Figure 3:**
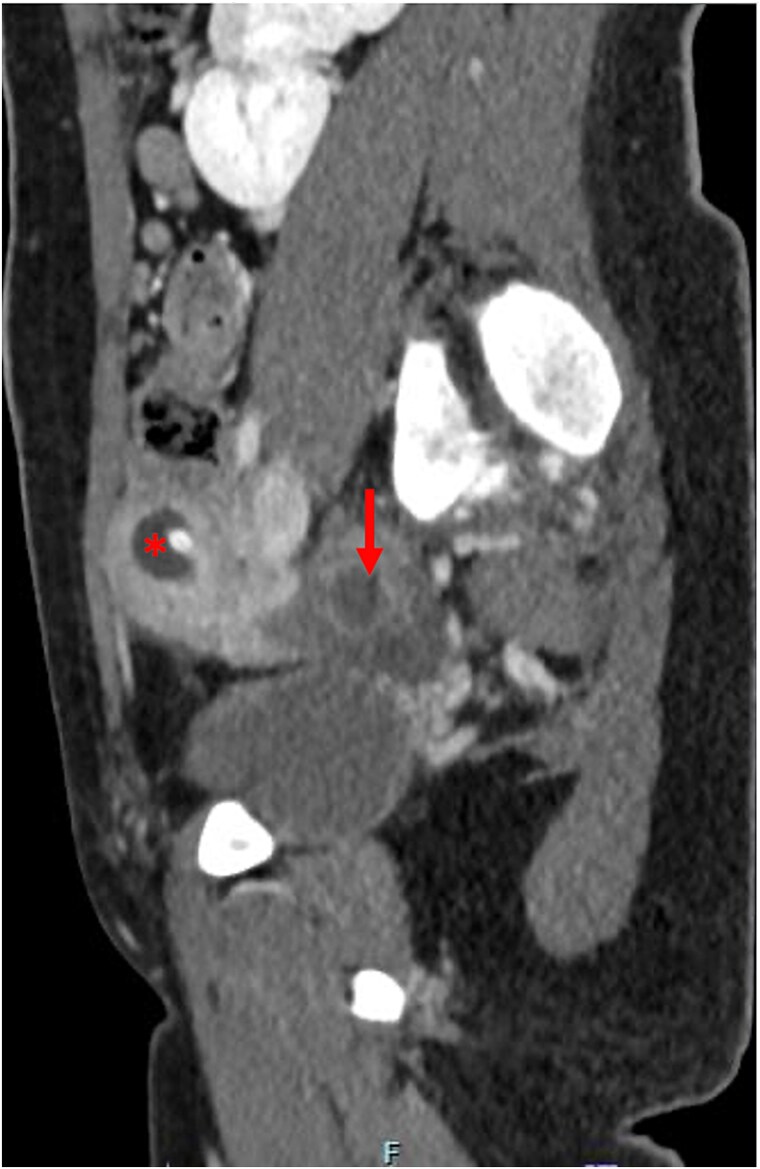
Post-operative computed tomography abdomen/pelvis demonstrating resolution of the hematometra (^*^) and improvement of the hematosalpinx (arrow).

The patient was discharged on the second post-operative day with the uterine drain. She presented for follow-up 2 weeks later with complete resolution of pain. A CT AP showed resolution of the hematometra and interval improvement in the hematosalpinx (Fig. 3). The patient was instructed to perform vaginal self-dilation using the Frank method for eventual uterovaginal reconstruction, and weekly flushing of the uterine drain to prevent clogging [4]. A gynecology outpatient consult was placed for pharmacologic management of ovulation.

## Discussion

We present a case of an adolescent girl with no significant past medical history who was found to have anatomy consistent with MRKH syndrome: a right-sided uterus with hematometra and ipsilateral hematosalpinx. She underwent laparoscopic uterine aspiration and drain placement for treatment of hematometra and hematosalpinx, with complete resolution of symptoms. To our knowledge, this is the first reported case of uterine drain placement to manage symptoms of and prevent future episodes of hematometra and hematosalpinx in a patient with MRKH syndrome.

It is rare for a patient with MRKH syndrome to have a functioning uterus with hematometra as our patient did. A cohort study demonstrated only 3% of patients with MRKH syndrome have uteri with radiographic signs of functioning endometrium, including intraluminal blood or adenomyosis [5]. Another cohort study similarly found only 2.6% of patients with MRKH syndrome to have a fully differentiated three-layer uterus with hematometra or ipsilateral hematosalpinx [2].

American College of Obstetrics and Gynecology guidelines recommend primary vaginal elongation by dilation as first-line of treatment for sexual function, rather than surgery [6]. Over 95% of patients who underwent dilation achieve successful lengthening of the vagina to allow for normal sexual function [7]. Of the 5% patients who are unsuccessful with or elect not to undergo vaginal dilation, surgical treatment may be considered. Multiple surgical approaches exist, including creation of a neovagina with gluteus skin flaps, peritoneal bridge, traction devices, and bowel interposition, with no consensus on the optimal surgical technique for best functional outcomes [6, 8].

A pelvic MRI is the imaging modality of choice for diagnosis of Mullerian duct anomalies [9]. When devising a reconstruction plan, surgeons must have a full understanding of the patient’s anatomy, such as through initial laparoscopy, since the full extent of complex anomalies may not be suspected even after imaging [10].

Our patient had a supernumerary right kidney. Cohort studies have demonstrated that 30%–60% of patients with MRKH syndrome have renal malformations, likely due to the close link between genital and urinary embryonic development [1, 11]. Among the patients with renal malformations, only 6%–12% have a duplex kidney like our patient [1, 11].

Our post-operative plan was to leave the uterine drain to facilitate menstrual flow with weekly flushing to prevent clogging. Future reconstructive surgery is planned, ideally using the patient’s own dilated vaginal canal. However, if sufficient vaginal elongation is not achieved, or complications occur—such as infection, drain dislodgment, or inability to replace the drain via a cutaneous fistula—intestinal interposition may be considered. Even a partial vaginal elongation would allow for a shorter intestinal segment to bridge the gap, preserving an external introitus, menstrual flow, and optimal sexual sensation and function.

## Conclusion

MRKH syndrome can present in adolescents as hematometra and hematosalpinx. In our patient, laparoscopic uterine drain placement resulted in the resolution of clinical symptoms and radiographic findings.
